# Long-Term Effectiveness of a Game-Based Mobile App for Training in Cardiopulmonary Resuscitation and Automated External Defibrillator Use: Nonrandomized Controlled Trial

**DOI:** 10.2196/78689

**Published:** 2026-05-06

**Authors:** Ruting Gu, Yueshuai Pan, Jing Han, Min Wang, Xiaomin Liu, Xia Liu, Qianqian Li, Jingyuan Wang, Shanshan Ji, Changfang Shi, Haiqing Zhou, Lili Wei

**Affiliations:** 1 Affiliated Hospital of Qingdao University, 16 Jiangsu Road, Qingdao, 266000, China, 1 18661807007, 1 053282911; 2 Qingdao University, Qingdao, China

**Keywords:** game-based, mobile app, cardiopulmonary resuscitation, automated external defibrillator, training

## Abstract

**Background:**

Bystander cardiopulmonary resuscitation (CPR) and automated external defibrillator (AED) use are critical for improving survival after out-of-hospital cardiac arrest. Although conventional training methods are initially effective, they are often hampered by rapid skill decay over time. Game-based mobile apps have emerged as a promising and scalable alternative for CPR and AED education; however, evidence of their long-term efficacy remains scarce.

**Objective:**

This study aimed to evaluate the integration of a game-based mobile app into traditional CPR and AED training. We assessed its impact on university students’ theoretical knowledge, practical skills, and theoretical knowledge retention, as well as their willingness to perform CPR and their awareness of disseminating these skills.

**Methods:**

A nonrandomized controlled trial was conducted among university students in China from March 21 to September 21, 2024. Participants were assigned to either an experimental group, which received game-based mobile app training supplemented with traditional training, or a control group, which received traditional training only. The game-based app featured a simulated scenario that required users to execute the correct sequence of resuscitation procedures and operate a virtual AED under time constraints. The intervention period lasted for 6 months. Participants’ theoretical knowledge and practical skills were assessed immediately after training (baseline) and at the 7-day follow-up. Long-term retention of knowledge, willingness to perform CPR, and dissemination awareness were evaluated at the 6-month follow-up. Data were analyzed using SPSS software (IBM Corp), employing the chi-square test, Mann-Whitney *U* test, and Wilcoxon signed-rank test.

**Results:**

A total of 481 participants completed the entire survey (n=241 in the experimental group and n=240 in the control group). In the short-term (7-d) assessment, the experimental group demonstrated significantly higher scores in both theoretical knowledge (*P*=.02) and practical skills (*P*<.001) compared to the control group. This advantage was maintained in the long term, with the experimental group showing superior knowledge retention at the 6-month follow-up (median score: 9/10 vs 8/10; *P*<.001). Furthermore, a majority of all participants expressed willingness to perform CPR on strangers (70.9%, 341/481) and to disseminate first-aid knowledge (92.1%, 443/481). However, no significant intergroup differences were observed for these latter 2 outcomes (*P*=.85 and *P*=.97, respectively).

**Conclusions:**

Despite the methodological limitations inherent in this nonrandomized study, our findings indicate that supplementing traditional training with the game-based mobile app significantly enhanced short-term acquisition of theoretical knowledge and practical skills and promoted sustained knowledge retention. This supports the app’s potential as an effective and promising complement to conventional CPR and AED training programs.

## Introduction

Out-of-hospital cardiac arrest (OHCA) is an important global public health problem with poor outcomes [1]. The worldwide incidence of OHCA among apparently healthy adults younger than 40 years of age ranges from 4 to 14 per 100,000 person-years [2]. Of an estimated 350,000 to 450,000 total annual OHCA cases in the United States, only approximately 10% survive [3]. Similarly, in Europe, the annual incidence of OHCA is between 67 and 170 per 100,000 inhabitants, with a mean survival-to-hospital discharge rate of 8% [4]. The crude incidence of emergency medical service–assessed OHCA in China is 95.7 per 100,000 population, with a low rate of resuscitation attempts (31.8%). The outcomes of emergency medical service–treated nontraumatic OHCA are unsatisfactory, with only 1.2% surviving to hospital discharge or 30 days [5]. However, the population in mainland China is approximately 1.41 billion [6], representing a substantial public health burden. Early bystander cardiopulmonary resuscitation (CPR) and rapid public access defibrillation are critical determinants of survival. Unfortunately, the bystander CPR rate in China is suboptimal compared with rates reported in the United States, Europe, and Japan [7,8]. One contributing factor may be the limited coverage and insufficient dissemination of bystander CPR training and retraining in China [9,10]. Therefore, extensive public education in basic life support is essential to improve OHCA outcomes [11].

Recently, technological advancements have become increasingly pivotal in promoting awareness and training for CPR and automated external defibrillator (AED) use [12]. Most studies focus on the initial acquisition and retention of CPR and AED knowledge and skills following training [13]. Initiatives such as the International Liaison Committee on Resuscitation World Restart a Heart Initiative are instrumental in fostering a culture of preparedness and encouraging widespread layperson CPR training, thereby helping to save countless lives [14]. Despite these efforts, key barriers—including uncertainty regarding correct CPR and AED procedures, fear of causing harm, and concerns about legal liability—continue to hinder public willingness to act during cardiac emergencies [15,16]. This underscores a critical need not only to raise public awareness and disseminate information on the importance of CPR and AED but also to develop and provide more innovative and accessible training methodologies [17].

In response to this need, we designed and developed a game-based mobile app for CPR and AED training. This study aimed to investigate whether integrating this app with traditional training could improve the acquisition and retention of CPR and AED knowledge and skills compared with traditional training alone. A nonrandomized controlled trial involving 481 participants from 2 Chinese universities was conducted to evaluate this hypothesis.

## Methods

### Study Design

We conducted a prespecified posttraining survey to compare the effectiveness of incorporating a game-based mobile app into traditional training methods on CPR and AED theoretical knowledge, practical skills, willingness, and dissemination awareness of CPR and AED among university students. The participants received unified theoretical and operational training. Participants in the experimental group used a game-based mobile app for simulation exercises, whereas those in the control group received no additional intervention. In this study, we strictly followed the Consolidated Standards of Reporting Trial (CONSORT)-EHEALTH (V 1.6.1) guidelines for reporting mobile health intervention trials, as detailed in Checklist 1.

### Participants

Using a convenience sampling method, 2 universities in the researcher’s city with comparable curricula were selected as research sites. Based on a natural grouping design, School A—which had similar curriculum systems, student compositions, and teaching resources—was assigned as the control group, while School B was designated as the experimental group. The inclusion criteria were as follows: adults aged 18 years or older, ownership of a smartphone (iOS or Android), and no prior participation in any CPR and AED training course. The exclusion criteria included physical limitations that would prevent the performance of CPR and AED techniques (including back or joint conditions).

### Ethical Considerations

This study was approved by the Medical Ethics Committee of the Affiliated Hospital of Qingdao University (QYFYWZLL29327). All participants took part voluntarily and provided written informed consent. Participants were informed of their right to withdraw at any time. To ensure privacy, all data were anonymized such that no individuals could be identified in any published results. In addition, no financial compensation was provided to participants.

### Features of the CPR and AED Gamification App

The gamified app was designed based on the CPR and AED procedure guidelines provided by the Red Cross Society of China Training Center. Game scenarios were developed accordingly by the research team. Software engineers used the Cocos2D game engine and the C++ programming language for development, which helped reduce costs while maintaining professional quality and achieving high visual realism in operational scenes. The app is compatible with multiple operating systems, including Windows, Android, and macOS, and can be installed on devices such as laptops, tablets, and smartphones via a downloadable installation package. This enables users to operate the app anywhere, even without an internet connection.

The CPR and AED gamified app is divided into 2 portals: a student portal and an administrator portal. The student portal comprises 4 modules: Personal Information, Operational Practice Guidance, Game Module, and System Settings. The administrator portal includes 2 modules: Information Review and Data Statistics. By integrating CPR and AED operational content with game elements, the app is designed to enhance student engagement and learning interest.

Students activated the game by registering with their mobile phone numbers. During gameplay, users interact directly with on-screen elements—such as the shoulder, neck, center of the chest, AED storage compartment button, and AED electrode pad switch—by clicking on the relevant parts and following prompts to complete each step. When moving objects, such as a battery or an electrode plug, users can either click the object and then the target location or drag the object directly to the desired position. Students were required to correctly perform the CPR and AED procedure within a specified time limit. The practice scenario consisted of 6 sequential stages that had to be completed in the correct order. Screenshots illustrating the practice process in the CPR and AED gamification app are provided in Multimedia Appendix 1 (screenshots of the CPR and AED gamification app).

1. Ensure scene safety
  - Approach the scene with caution and confirm that the environment is safe for both rescuers and the person.
2. Check for responsiveness
  - Assess responsiveness by tapping the person’s shoulders and shouting (eg, “Are you okay?"). If no response is obtained, immediately check the person’s breathing.
3. Check breathing
  - Check for absence of breathing or only gasping while simultaneously checking the carotid pulse. Confirm whether a pulse is definitely palpable within 10 seconds.
4. Activate emergency response system and retrieve AED
  - Send someone for help and to retrieve an AED. If alone, activate the emergency response system (eg, call the local emergency number) and get the AED if immediately available.
5. Begin high-quality CPR: follow the C-A-B sequence
  - C—Chest compressions
    - Position: Heel of one hand on the center of the chest.
    - Technique: Arms straight, shoulders directly over the hands.
    - Depth: 5 to 6 cm.
    - Rate: 100 to 120 compressions per minute.
    - Recoil: Allow complete chest recoil.
    - Minimize interruptions in compressions.
  - A—Open the airway
    - After 30 compressions, open the airway using the head-tilt or chin-lift maneuver.
  - B—Rescue breaths
    - Give 2 breaths, each delivered over about 1 second, ensuring visible chest rise.
    - Compression-to-ventilation ratio: 30:2.
    - Note: If unable or unwilling to give rescue breaths, perform compression-only CPR.
6. Early defibrillation with AED
  - Once the AED arrives:
    - Turn on the device and attach the electrode pads to the person’s bare chest.
    - Ensure that no one is touching the person during rhythm analysis.
    - If a shock is advised, clear all individuals from contact with the person and deliver the shock promptly.
    - Resume CPR immediately after shock delivery, beginning with chest compressions.
    - Continue repeated cycles of approximately 2 minutes of CPR followed by AED rhythm analysis as instructed.

### Intervention

This study was conducted between March 21 and September 21, 2024. The training sessions were delivered by instructors certified by the Red Cross Society of China. To ensure standardization, all instructors first underwent a researcher-led briefing to ensure a comprehensive understanding of the CPR and AED program and its key learning objectives. On March 21, 2024, a 30-minute theoretical training session on CPR and AED was conducted, followed by a theoretical test. Subsequently, a 30-minute practical demonstration was performed by the researchers in accordance with the standard procedure. Each instructor was assigned to train 10 students, allowing 1 hour for independent practice and question-and-answer interaction. Finally, a training supervisor carried out the skill assessment based on unified evaluation criteria.

Subsequently, the researchers provided the installation package to participants in the experimental group and ensured that each student successfully completed the download and installation process. They also explained the app’s usage procedures and module functions to ensure that all students were familiar with its features. Students were permitted to use the game program freely at any time over the following 6 months, without needing to perform any additional operations. Each student was required to spend a cumulative duration of at least 30 minutes per week using the app and to complete at least 1 in-game level during that period. If a student failed to meet the level-completion requirement, the instructor was responsible for identifying the reasons and providing relevant guidance—addressing difficulties and questions raised during gameplay—until the student mastered the corresponding knowledge point and successfully passed the level. Instructors could access students’ learning data by searching for their names directly through the management portal.

No additional intervention was provided to the control group during the study period. However, these participants were offered access to the game-based mobile app after they completed the postintervention survey.

### Data Collection

Prior to the commencement of the study, all data collectors and CPR and AED skill assessors received standardized training from the investigators. An electronic questionnaire was administered immediately after the theoretical training session to evaluate participants’ knowledge acquisition. Following practical skill training, participants were invited in batches to a unified testing center for CPR and AED skills assessment. Assessors were explicitly instructed that they would evaluate participants from any study group and were required not to inquire about or speculate on group assignments throughout the process. In line with the study protocol and consistent with the structure of our team’s first-aid courses at the university, both theoretical and practical tests were repeated 7 days later. Six months after completing the CPR and AED training, all participants were asked to complete a follow-up electronic questionnaire. This survey aimed to compare the 2 training methods in terms of long-term retention of theoretical knowledge, willingness to perform CPR, and dissemination of CPR-AED awareness.

### Questionnaires

The study used several questionnaires. First, a general information questionnaire was used to collect demographic data, including age, sex, and major. Second, the CPR and AED Theoretical Knowledge Questionnaire, developed by the Red Cross Society of China, was used. This instrument comprises 10 items covering key aspects of CPR and AED application, with total scores ranging from 0 to 100. The next section assessed participants’ attitudes and willingness to perform CPR and AED in cardiac arrest scenarios. The Willingness to Perform CPR and AED Questionnaire was developed by the research team based on a review of relevant literature [18,19], and the final version contained 6 items. Additionally, participants completed a modified version of the Dissemination of CPR Awareness Questionnaire, originally developed by Nas et al [20]. This modified instrument included 4 items specifically designed to evaluate the dissemination of CPR and AED awareness and was used to assess participants’ knowledge sharing in this domain.

### Sample Size and Power

The sample size calculation was based on a previous study [21], which reported significant differences in CPR compression depth and frequency compliance rates between a VR training group and a traditional training group (51% vs 75%; 50% vs 63%). Using these proportions (*p*_₁_=0.50, *p*_₂_=0.63), a 2-sided *α* of .05, and a statistical power (1 – *β*) of 80% (*β*=.20), the calculated sample size was 227 participants per group. To account for a potential 5% invalid response or attrition rate, the minimum required sample size was adjusted to 239 per group. Accordingly, this study enrolled 241 participants in the control group and 240 in the experimental group.

The formula used to calculate the sample size is as follows:


(1) $$N=\frac{{\left(Z_{1-\alpha /2}\sqrt{2\bar{p}(1-\bar{p})}+Z_{1-\beta }\sqrt{p_{1}(1-p_{1})+p_{2}(1-p_{2})}\right)}^{2}}{{\left(p_{1}-p_{2}\right)}^{2}}$$

### Statistical Analysis

All statistical analyses were performed using SPSS version 27.0 (IBM Corp). Descriptive statistics were presented as means with SDs or as numbers and percentages, as appropriate. The normality of the data distribution was assessed using the one-sample Kolmogorov-Smirnov test. Since the skill scores deviated significantly from a normal distribution (*df*=481, *P*<.001), nonparametric tests were used for subsequent comparisons. The Mann-Whitney *U* test was used to compare outcomes between the 2 independent groups, whereas the Wilcoxon signed-rank test was applied for within-group comparisons across different time points. Categorical variables, expressed as numbers and percentages, were compared using the chi-square test or Fisher exact test, depending on the expected frequencies. All *P* values were 2-sided, and statistical significance was set at *P*<.05.

## Results

### Analytical Sample

**Figure 1.**
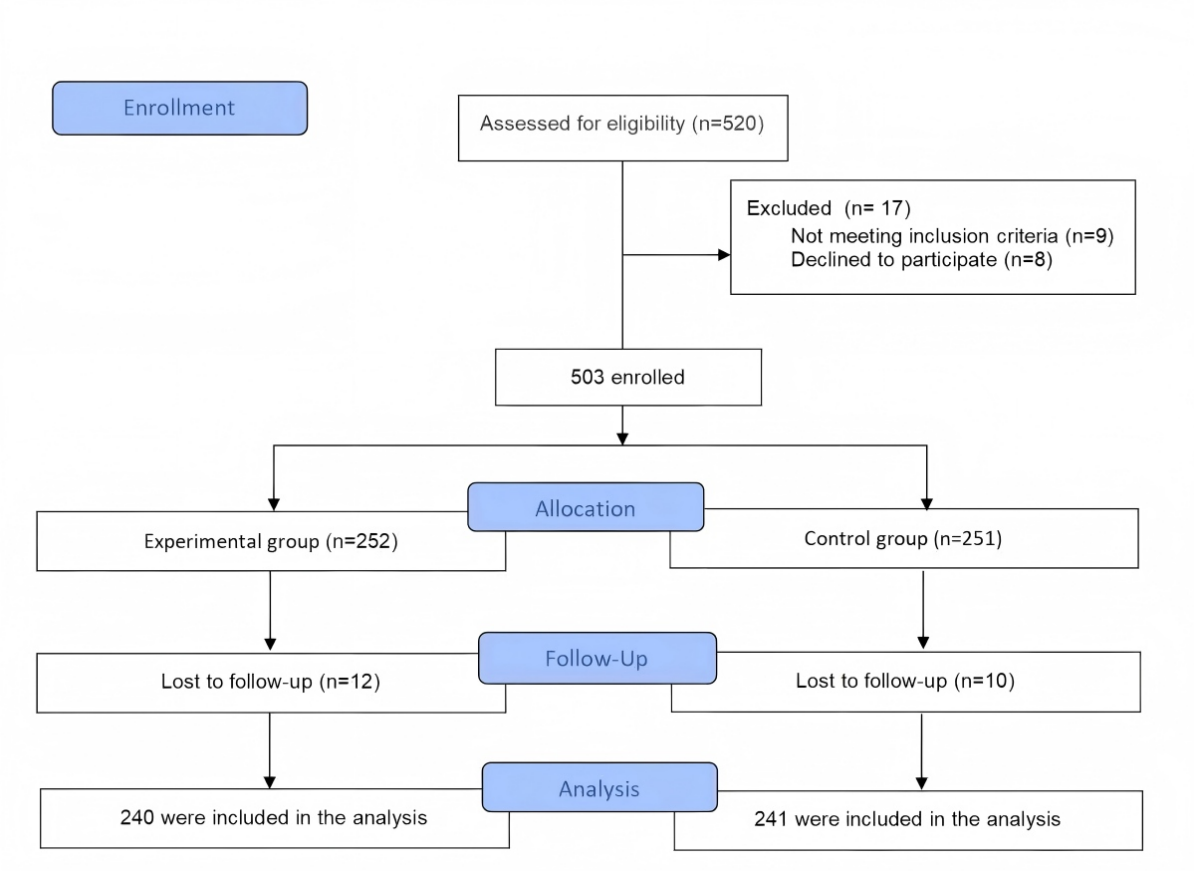
Flowchart of included participants.

A total of 503 participants were enrolled in this study (n=252 in the experimental group and n=251 in the control group). After the 6-month intervention, which included 2 follow-up assessments, 4.8% (12/252) of participants in the experimental group and 4% (10/251) of participants in the control group were lost to follow-up or withdrew. Consequently, the final analysis included 240 and 241 participants in the experimental and control groups, respectively (Figure 1).

### Participant Characteristics

Table 1 presents the baseline characteristics of the participants. A total of 481 university students were included in this study, comprising 253 men and 228 women, with a median age of 20 (IQR 19-20) years. Regarding academic major, the proportion of medical students was comparable between the control group (38/241, 15.8%) and the experimental group (32/240, 13.3%). No statistically significant differences were observed in any baseline characteristics between the 2 groups (all *P*>.05).

**Table 1.** Participants’ characteristics at baseline.

Variable	Total (N=481)	Control group (n=241)	Experimental group (n=240)	Z/*χ*^2^ (*df*)*^a^*	*P* value
Age (y), median (IQR)	20 (1)	20 (1)	20 (1)	1.598	.11
Sex, n (%)				0.051 (1)	.82
Female	228 (47.4)	113 (46.9)	115 (47.9)		
Male	253 (52.6)	128 (53.1)	125 (52.1)		
Major, n (%)				0.573 (1)	.45
Medical specialty	70 (14.6)	38 (15.8)	32 (13.3)		
Nonmedical specialty	411 (85.4)	203 (84.2)	208 (86.7)		

a (df) indicates the degrees of freedom of the chi-square test.

### Theoretical and Practical Skills Tests

At baseline, no significant differences were found in the theoretical and practical skills tests for CPR and AED between the control and experimental groups (*P*=.50 and *P*=.63, respectively; Tables 2 and 3). After 7 days, the experimental group achieved significantly higher scores than the control group in both theoretical knowledge and practical skills (*P*=.02 and *P*<.001, respectively).

**Table 2.** Difference in theoretical scores between the control and experimental groups.^a^

Time point	Control group, median (IQR)	Experimental group, median (IQR)	Z	*P* value
First theoretical scores	90 (10)	90 (10)	0.678	.50
7-day theoretical scores	80 (10)	90 (10)	2.323	.02

a Within-group comparisons (Wilcoxon signed-rank test): control group: *Z*=−6.214, *P*<.001; experimental group: *Z*=−6.9, *P*<.001.

**Table 3.** Difference in skill performance scores between the control and experimental groups.^a^

Time point	Control group, median (IQR)	Experimental group, median (IQR)	Z	*P* value
First practical skill scores	84 (6)	85 (8)	0.481	.63
7-day practical skill scores	84 (7)	87 (4)	7.703	＜.001

a Within-group comparisons (Wilcoxon signed-rank test): control group: *Z*=−1.241, *P*=.22; experimental group: Z=5.261, *P*<.001.

In the control group, the theoretical score significantly decreased from the immediate posttest to the 7-day follow-up (*P*<.001; Table 2). In contrast, the observed decline in operational skills was not statistically significant (*P*=.22; Table 3).

In the experimental group, theoretical scores exhibited a statistically significant change between the immediate posttest and the 7-day follow-up (*Z*=−6.9, *P*<.001). Although the median scores remained constant at 90, the distribution of the data changed. This indicates a substantial redistribution in knowledge retention that is not reflected by the median alone (Table 2). Furthermore, practical skills demonstrated a significant improvement at the 7-day follow-up compared with the initial assessment (*P*<.001; Table 3).

### Theoretical Knowledge Retention

The responses to individual CPR and AED theoretical knowledge questions are detailed in Table 4. The experimental group achieved a significantly higher total theoretical knowledge score than the control group (9 vs 8 points; *P*<.001). Notably, the experimental group also demonstrated superior performance on several key items: a higher proportion of the experimental group correctly identified the initial step as assessing scene safety (204/240, 85% vs 184/241, 76.3%; *P*=.02), the correct chest compression rate (200/240, 83.3% vs 174/241, 72.2%; *P*=.003), the appropriate compression depth (186/240, 77.5% vs 166/241, 68.9%; *P*=.03), and the correct compression-to-ventilation ratio (189/240, 78.8% vs 169/241, 70.1%; *P*=.03). No significant differences were found for the remaining items (Table 4).

**Table 4.** Theoretical knowledge retention.

Questions and answers	Participants, n (%)			*χ*^2^ (*df*)*^a^*	*P* value^b^
	All (N=481)	Control group (n=241)	Experimental group (n=240)		
Q1. When you see a person lying on the ground, what do you do first?				5.771 (1)	.02
Correct: Check for safety	388 (80.7)	184 (76.3)	204 (85)		
Incorrect	93 (19.3)	57 (23.7)	36 (15)		
Check for response	—^c^	26 (10.8)	17 (7.1)		
Check for breathing	—	31 (12.9)	19 (7.9)		
Q2. The golden time for rescuing person with cardiac arrest is:				0.806 (1)	.37
Correct: 4-6 min	433 (90)	214 (88.8)	219 (91.3)		
Incorrect	48 (10)	27 (11.2)	21 (8.7)		
7-8 min	—	22 (9.1)	19 (7.9)		
More than 10 min	—	5 (2.1)	2 (0.8)		
Q3. To check if the person is conscious, you have to do the following:				0.460 (1)	.50
Correct: Gently shake his (her) shoulders and ask loudly, “Sir (lady) can you hear me?”	422 (87.7)	209 (86.7)	213 (88.8)		
Incorrect	59 (12.3)	32 (13.3)	27 (11.2)		
Gently shake his (her) shoulders, pinch his arm, and scream, “Sir (lady) can you hear me?”	—	22 (9.1)	18 (7.5)		
Gently shake his head and ask loudly, “Sir (lady) can you hear me?”	—	10 (4.2)	9 (3.7)		
Q4. How fast should you perform chest compressions on adults?				8.619 (1)	.003
Correct: 100-120 beats per minute	374 (77.8)	174 (72.2)	200 (83.3)		
Incorrect	107 (22.2)	67 (27.8)	40 (16.7)		
60-80 beats per minute	—	27 (11.2)	21 (8.8)		
80-100 beats per minute	—	40 (16.6)	19 (7.9)		
Q5. How deep should you perform chest compressions when performing CPR^h^?				4.553 (1)	.03
Correct: 5-6 cm	352 (73.2)	166 (68.9)	186 (77.5)		
Incorrect	129 (26.8)	75 (31.1)	54 (22.5)		
About 4 cm	—	39 (16.2)	31 (12.9)		
About 5 cm	—	36 (14.9)	23 (9.6)		
Q6. Which of the following is not the method of chest compression?				0.075 (1)	.78
Correct: Use the hip joint as the fulcrum and press vertically downward with the strength of the shoulder	344 (71.5)	171 (71)	173 (72.1)		
Incorrect	137 (28.5)	70 (29)	67 (27.9)		
Overlap the bases of the 2 palms	—	34 (14.1)	27 (11.2)		
The wrist, elbow, and shoulder joints are in a straight line without any curvature	—	36 (14.9)	40 (16.7)		
Q7. The ratio of chest compression to artificial respiration on adult is:				4.701 (1)	.03
Correct: 30:2	358 (74.4)	169 (70.1)	189 (78.8)		
Incorrect	123 (25.6)	72 (29.9)	51 (21.2)		
15:1	—	43 (17.9)	28 (11.6)		
15:2	—	29 (12)	23 (9.6)		
Q8. When should you stop chest compressions to give mouth-to-mouth ventilations?				0.438 (1)	.51
Correct: After 30 compressions	385 (80)	190 (78.8)	195 (81.3)		
Incorrect	96 (20)	51 (21.2)	45 (18.7)		
After 10 compressions	—	28 (11.6)	20 (8.3)		
After about 1 min of compressions	—	23 (9.6)	25 (10.4)		
Q9. When rescuing a person with cardiac arrest, if someone brings in an AED^e^, what should the rescuer do?				3.418 (1)	.06
Correct: Persist in chest compression, after the AED is installed, defibrillation should be carried out quickly	391 (81.3)	188 (78)	203 (84.6)		
Incorrect	90 (18.7)	53 (22)	37 (15.4)		
To increase the success rate of defibrillation, chest compressions for about 2 min should be performed before electric shock, followed by rapid defibrillation	—	35 (14.5)	28 (11.7)		
Suspend chest compressions and prioritize the installation and use of AED	—	18 (7.5)	9 (3.7)		
Q10. How do you place the AED pads (Figure 2)?				3.025 (1)	.08
Correct: Figure 2C	418 (86.9)	203 (84.2)	215 (89.6)		
Incorrect	63 (13.1)	38 (15.8)	25 (10.4)		
Figure 2A	—	17 (7.1)	14 (5.8)		
Figure 2B	—	21 (8.7)	11 (4.6)		
Overall					
Total score (each correctly answered question is 1 point)^f^	—	8 (2)	9 (2)	4.034	<.001

a (df) indicates the degrees of freedom of the chi-square test.

b The chi-square test of independence was used to examine the association between group membership (experimental vs. control) and response outcome (correct vs. incorrect). No distinction was made among different types of errors.

c Not applicable.

d CPR: cardiopulmonary resuscitation.

e AED: automated external defibrillator.

f Total scores are median values (IQRs).

**Figure 2.**
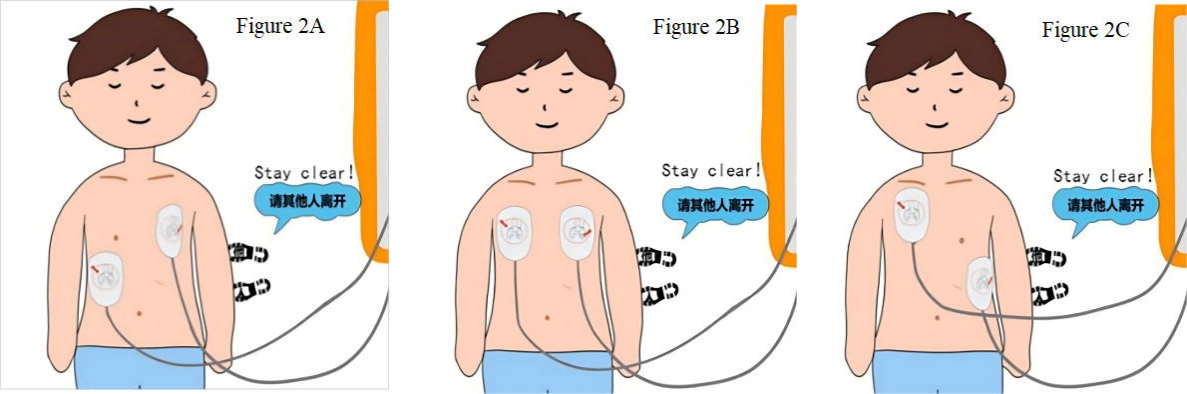
Position for placing the automated external defibrillator pads.

### Willingness to Perform CPR and AED

Participants’ self-reported willingness to perform CPR and use an AED is summarized in Table 5. Overall, 34.1% (164/481) of participants felt capable of performing both CPR and using an AED. Of these participants, 29.9% (144/481) attributed this capability directly to the training received in the study, and this proportion did not differ significantly between groups (*P*=.53). When asked about assisting a stranger, a strong majority of participants (341/481, 70.9%) expressed willingness, with no significant difference observed between the control group (168/241, 69.7%) and the experimental group (173/240, 72.1%) (*P*=.85).

**Table 5.** Self-reported attitude and willingness.

Question and answers	Participants, n (%)			*χ*^2^ (*df*)^a^	*P* value
	All (N=481)	Control group (n=241)	Experimental group (n=240)		
1. Do you feel capable to perform CPR^b^ after study participation?				1.270 (2)	.53
Yes, because of study participation	164 (34.1)	88 (36.5)	76 (31.7)		
Yes, because I did receive CPR training after the study	144 (29.9)	69 (28.6)	75 (31.2)		
No	173 (36)	84 (34.9)	89 (37.1)		
2. If you were to witness a cardiac arrest of a stranger, would you start CPR?				0.335 (2)	.85
Yes	341 (70.9)	168 (69.7)	173 (72.1)		
No	81 (16.8)	31 (12.9)	28 (11.7)		
Do not know	59 (12.3)	42 (17.4)	39 (16.2)		
3. If you were to witness a cardiac arrest of a family member or friend, would you start CPR?				0.220 (2)	.90
Yes	461 (95.8)	232 (96.3)	229 (95.4)		
No	11 (2.3)	5 (2)	6 (2.5)		
Do not know	9 (1.9)	4 (1.7)	5 (2.1)		
4. I have the feeling that I am capable of helping somebody experiencing cardiac arrest				0.643 (4)	.96
Strongly agree	71 (14.7)	34 (14.1)	37 (15.4)		
Agree	200 (41.6)	101 (41.9)	99 (41.3)		
Neutral	106 (22)	55 (22.8)	51 (21.3)		
Disagree	72 (15)	34 (14.1)	38 (15.8)		
Strongly disagree	32 (6.7)	17 (7.1)	15 (6.2)		
5. Can you accurately recall and apply CPR and AED^c^ skills in an emergency?				0.803 (4)	.94
Definitely	61 (12.7)	29 (12)	32 (13.3)		
Basically	188 (39.1)	93 (38.6)	95 (39.6)		
Neutral	139 (28.9)	73 (30.3)	66 (27.5)		
Unlikely	48 (10)	25 (10.4)	23 (9.6)		
Impossible	45 (9.3)	21 (8.7)	24 (10)		
6. I would be scared to perform CPR				0.167 (1)	.68
Yes	202 (42)	99 (41.1)	103 (42.9)		
No	279 (58)	142 (58.9)	137 (57.1)		

a (df) indicates the degrees of freedom of the chi-square test.

b CPR: cardiopulmonary resuscitation.

c AED: automated external defibrillator.

The vast majority of participants (461/481, 95.8%) reported that they would perform CPR and use an AED on a relative or friend, and no significant difference was found between the control group (232/241, 96.3%) and the experimental group (229/240, 95.4%) (*P*=.90). Overall, 56.3% (271/481) of participants felt capable of performing these skills, and 51.8% (249/481) reported confidence in recalling and applying first-aid skills when needed; neither measure differed significantly between groups (*P*=.96 and *P*=.94, respectively). In contrast, 42% (202/481) of participants reported fear of performing CPR and using an AED, with comparable levels between the control group (99/241, 41.1%) and experimental group (103/240, 42.9%) (*P*=.68).

### Dissemination of Awareness

The willingness to disseminate first-aid knowledge was high among participants, with 92.1% (443/481) expressing this intention.

Overall, 66.3% (319/481) of participants indicated that they would tell their family or friends about the importance of CPR, and this proportion did not differ significantly between the control group (66.8%, 161/241) and the experimental group (65.8%, 158/240) (*P*=.85). Similarly, 61.3% (295/481) of participants had already communicated with relatives or friends about the importance of CPR and AED training, with comparable rates between the control group (147/241, 61%) and the experimental group (148/240, 61.7%) (*P*=.94).

In total, 7.5% (36/481) of participants reported that their family members or acquaintances took concrete action, such as searching for CPR and AED training information, applying for a course, or attending a course. Although the proportion was higher in the experimental group (20/240, 8.3%) than in the control group (16/241, 6.6%), this difference was not statistically significant (*P*=.77). Detailed responses regarding the dissemination of CPR and AED awareness are provided in Table 6.

**Table 6.** Dissemination of cardiopulmonary resuscitation and automated external defibrillator awareness.

Question and answers	Participants, n (%)			*χ*^2^ (*df*)^a^	*P* value
	All (N=481)	Control group (n=241)	Experimental group (n=240)		
1. Would you be willing to spread knowledge related to CPR^b^ and AED^c^ skills to others?				0.253 (3)	.97
Strongly agree	364 (75.7)	183 (75.9)	181 (75.4)		
Agree	79 (16.4)	39 (16.2)	40 (16.7)		
Neutral	33 (6.9)	17 (7.1)	16 (6.7)		
Disagree	5 (1)	2 (0.8)	3 (1.2)		
2. Did you tell family or acquaintances about the importance of CPR and AED?				0.274 (2)	.87
Do not know	32 (6.7)	17 (7.1)	15 (6.3)		
No	130 (27)	63 (26.1)	67 (27.9)		
Yes	319 (66.3)	161 (66.8)	158 (65.8)		
3. Did you tell family or acquaintances about the importance of CPR and AED training?				0.318 (2)	.85
Do not know	29 (6.1)	16 (6.6)	13 (5.4)		
No	157 (32.6)	78 (32.4)	79 (32.9)		
Yes	295 (61.3)	147 (61.0)	148 (61.7)		
4. The reaction of your family members or friends to your participation in the emergency rescue training				0.517 (2)	.77
Do not know	230 (47.8)	117 (48.5)	113 (47.1)		
No response	215 (44.7)	108 (44.9)	107 (44.6)		
Positive reaction	36 (7.5)	16 (6.6)	20 (8.3)		

a (df) indicates the degrees of freedom of the chi-square test.

b CPR: cardiopulmonary resuscitation.

c AED: automated external defibrillator.

## Discussion

### Principal Findings

Integration of the game-based mobile app into training led to greater short-term mastery of both theoretical knowledge and practical CPR and AED skills compared with traditional methods. These findings are consistent with our previous research on other skill training [22] and are supported by preliminary studies indicating that gamification enhances learning outcomes in CPR training [23,24]. The app integrates game design elements into animated procedural simulations, which likely helps maintain learner engagement. Furthermore, its accessibility on mobile phones and computers allows for flexible, repeated practice in various settings. While these features position gamified mobile learning as a flexible, practical, and scalable tool—particularly when integrated with high-fidelity simulation—further rigorously designed studies are needed to confirm its long-term effectiveness and potential for optimizing CPR and AED training.

The follow-up assessment prioritized the evaluation of CPR and AED knowledge retention over practical skills, taking into account some participants’ upcoming internships or graduation. Results from the 6-month follow-up indicated a high level of knowledge retention, which is consistent with the findings reported by Nas et al [20]. In this study, the experimental group achieved marginally higher scores across the 10 CPR and AED knowledge items, and this difference was statistically significant. This outcome may be attributed to the game-based app’s emphasis on key procedural elements through a clear and visually engaging interface, which likely facilitates comprehension and long-term memory encoding. These observations suggest that a gamified mobile app could play a valuable role in supporting the sustained acquisition of CPR and AED knowledge. It is widely recognized that CPR and AED practical skills are prone to decay over time—a concern highlighted in the European Resuscitation Council Guidelines 2021 [25], which emphasize the need for regular retraining to sustain competency. These guidelines recommend distributing resuscitation training over time and maintaining competencies through frequent retraining. In this context, game-based mobile apps, given their accessibility and self-paced format, could effectively complement traditional training approaches, potentially extending reach and supporting ongoing skill maintenance.

Willingness to perform CPR and use an AED was generally high in both study groups. Compared with the control group, a greater proportion of participants in the experimental group expressed willingness to initiate CPR and AED procedures, and a slightly higher percentage reported feeling capable of assisting a person with cardiac arrest. This may be attributed to the game-based mobile app, which enabled repeated, low-stakes practice in diverse settings, thereby enhancing learners’ familiarity and self-efficacy. The self-paced learning feature likely reduced anxiety and fostered incremental confidence—advantages not typically offered by single-session training. Furthermore, the gamified environment may have contributed to a more engaging learning experience, potentially reducing the perceived dread and complexity associated with emergency scenarios [26]. Additionally, the app’s design, which breaks down the rescue process into clear, sequential steps with immediate feedback, may have helped users develop a structured mental model for responding in an actual cardiac arrest situation. Such clarity could alleviate the fear of making errors, a commonly reported barrier among lay responders [27]. However, the specific mechanisms underlying these effects require confirmation through studies specifically designed to establish causality. Notably, over 42% of participants across the study reported fear of performing CPR and using an AED, primarily due to concerns about causing secondary harm and facing legal consequences. These are widely documented barriers among laypersons, as also reflected in the study by Daud et al [15]. Together, these findings underscore that improving bystander willingness to act remains a critical challenge for future training initiatives.

Bystander-initiated CPR and rapid public access defibrillation are known to significantly improve survival outcomes after OHCA, with their combination exhibiting a synergistic effect [28]. A primary goal of resuscitation education for the public is, therefore, to raise awareness, train as many laypersons as possible, and develop innovative strategies to save more lives [29]. As is well known, the most common location for OHCA is the home or residence [30]; therefore, training that influences household members is of particular importance. In the present study, over 60% of the participants reported sharing information on the importance of CPR and AED with their family members or friends, indicating a potential diffusion of training awareness beyond the immediate learners. Some of these family members or friends took further active steps, such as searching for related materials, watching demonstration videos, or enrolling in formal courses, in response to participants’ encouragement.

This gamified mobile app was developed based on standardized CPR and AED training scenarios. Unlike traditional approaches such as on-site training or video-based instruction, it offers more flexible and accessible learning opportunities. The app incorporates game-like elements to promote interactive and intuitive engagement, as well as active participation among learners and their peers [31]. It also enables repeated, self-directed practice in diverse environments, which may strengthen familiarity with operational procedures. Furthermore, by breaking down the rescue process into sequential steps supported by real-time feedback, the app helps users establish a clear and actionable mental model for responding to an actual cardiac arrest. We propose that integrating this gamified CPR and AED training tool with conventional methods could serve as an effective complementary strategy for broadening the dissemination of CPR and AED knowledge and skills.

### Limitations and Future Study

This study has several limitations. First, the use of a nonrandomized controlled trial design represents a key methodological constraint. The lack of random assignment to the experimental or control group may have introduced selection bias, thereby limiting the strength of causal inferences that can be drawn. Future research should employ randomized controlled trials to validate these findings. Second, although high theoretical knowledge retention was observed, the study did not assess the retention of practical skills. Thus, it remains unclear how well retained knowledge translates into actual CPR and AED performance in real-world settings. Subsequent studies should incorporate longitudinal evaluations of both knowledge and hands-on competencies in simulated or real cardiac arrest scenarios. Third, the absence of a preintervention survey prevented comparisons between baseline and postintervention outcomes, which similarly applies to measures such as willingness to perform CPR and dissemination of awareness. Finally, the generalizability of the findings may be limited by the study population, which consisted predominantly of young, highly educated participants with relatively high information technology literacy. The effectiveness of this game-based mobile app may vary among older adults, individuals with lower digital literacy, or those in different cultural settings. Future studies involving more diverse populations are needed to assess the broader applicability and effectiveness of this approach.

### Conclusion

This study evaluated the integration of a game-based mobile app with traditional CPR and AED training. We assessed participants’ short-term acquisition of theoretical knowledge and practical skills, as well as long-term knowledge retention, willingness to act, and dissemination awareness after 6 months. The results indicate that supplementing traditional training with the game-based app may improve short-term mastery of both knowledge and practical skills and sustain knowledge retention over 6 months. Overall willingness to perform CPR and disseminate related knowledge was high, with no significant differences between groups. Despite the methodological limitations inherent in this nonrandomized study, the findings indicate that this accessible, interest-stimulating mobile learning tool may serve as a useful complement to existing CPR and AED training paradigms.

## Supplementary material

10.2196/78689 Multimedia Appendix 1 Screenshots of the cardiopulmonary resuscitation and automated external defibrillator gamification application.

10.2196/78689 Checklist 1 CONSORT-eHEALTH checklist (V 1.6.1).

## Abbreviations

AED: automated external defibrillator
CONSORT: Consolidated Standards of Reporting Trials
CPR: cardiopulmonary resuscitation
OHCA: out-of-hospital cardiac arrest
