# Supplementary material for: Long-Term Effectiveness of a Game-Based Mobile App for Training in Cardiopulmonary Resuscitation and Automated External Defibrillator Use: Nonrandomized Controlled Trial
Source: JMIR Mhealth Uhealth. 2026 May 6;14:e78689. doi: 10.2196/78689 (PMC13148323; doi:10.2196/78689)
Supplement: Multimedia Appendix 1 [file mhealth-v14-e78689-s001.docx]

CPR and AED Gamification Application

- **Logon Screen**


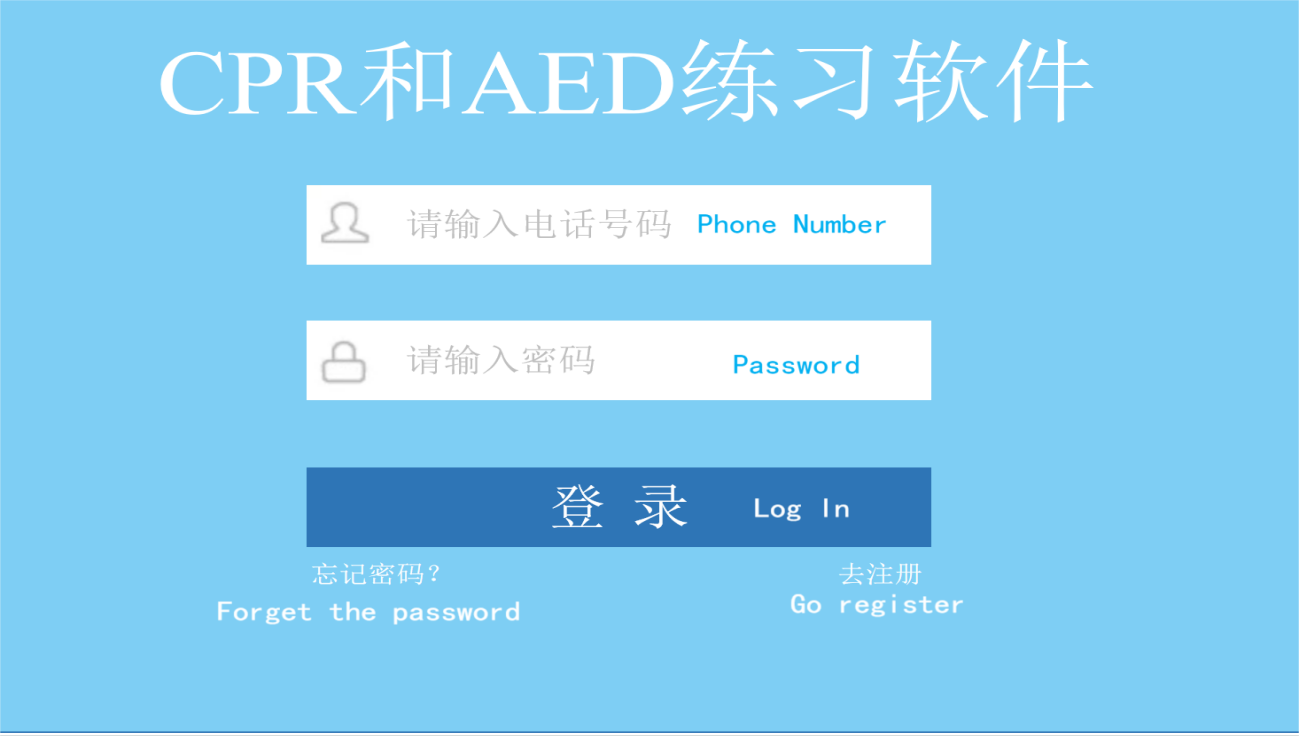


- **Onboarding tutorial**


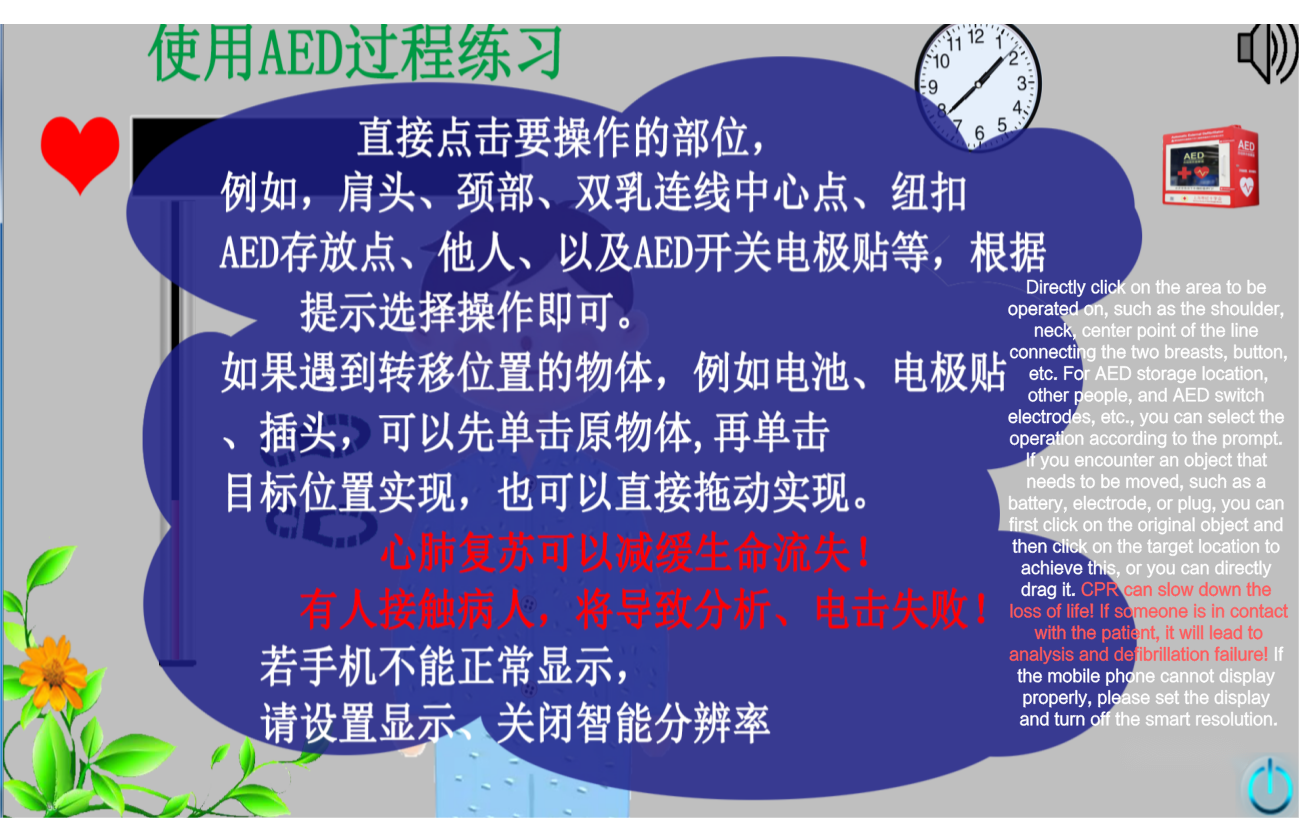


- **Practice process**

**Ensure Scene Safety and Check for responsiveness**

**
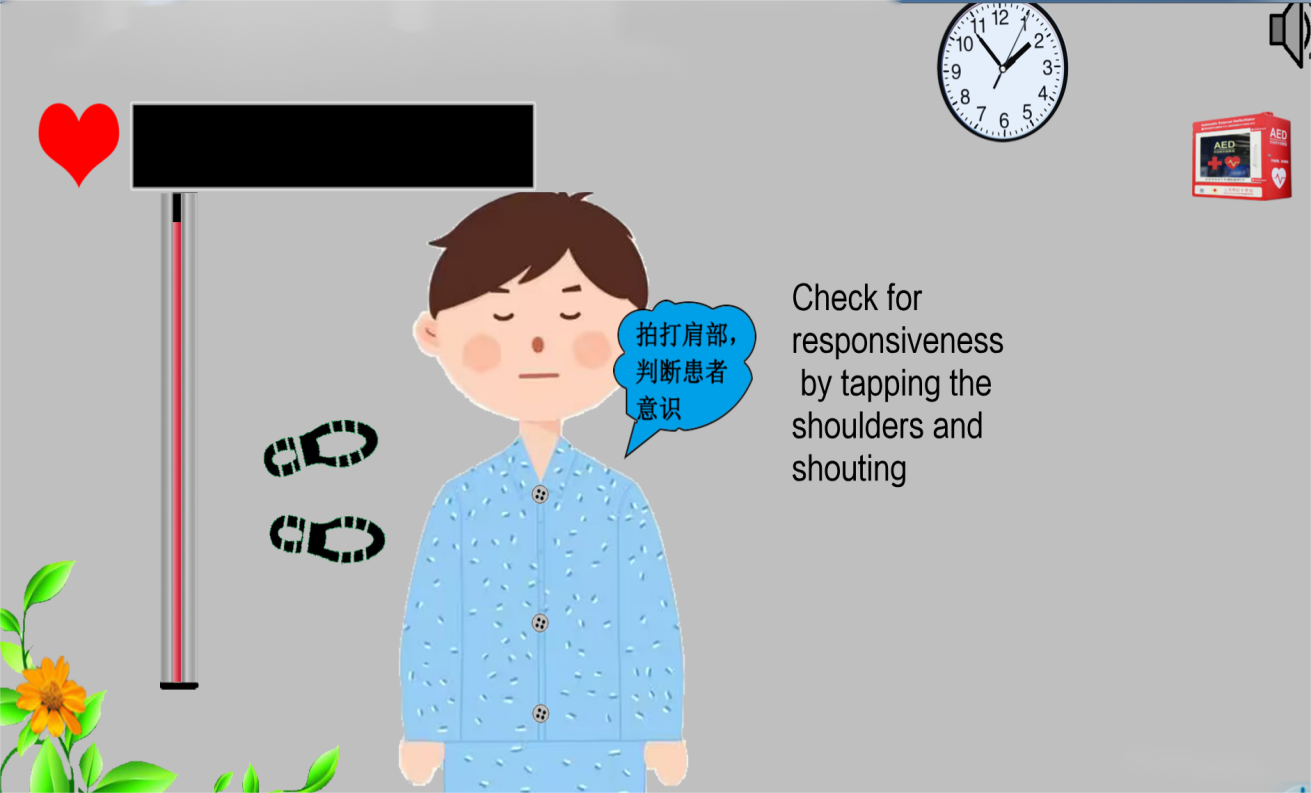
**

**Check the breathing**

**
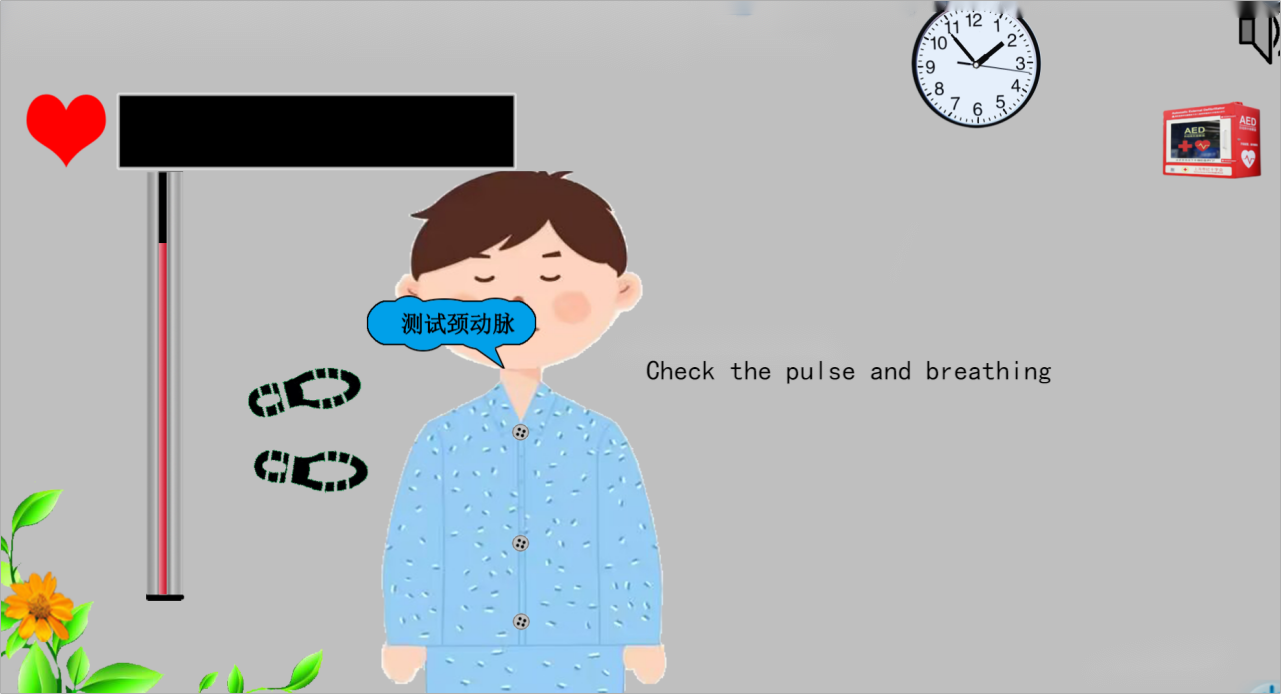
**

**Activate Emergency Response System and Retrieve AED**

**
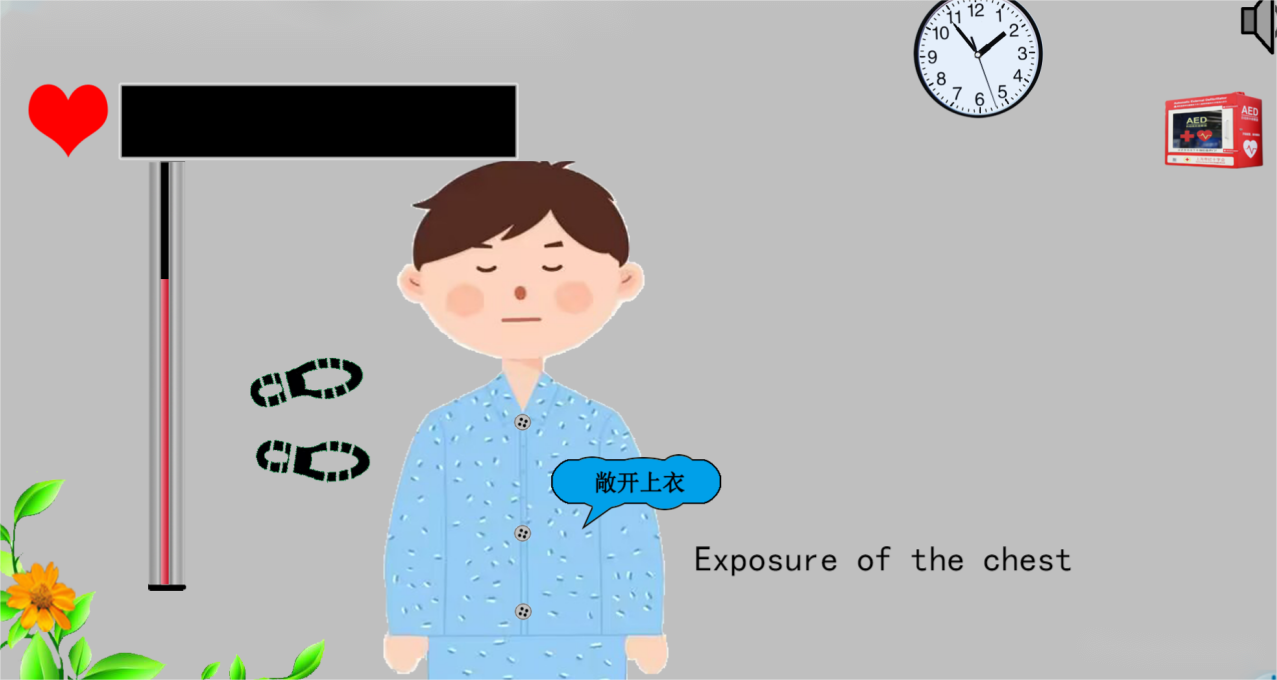
**

**Begin High-Quality CPR: Follow the C-A-B Sequence (C: Chest Compressions,**  **A: Open the Airway; B - Rescue Breaths)**

**
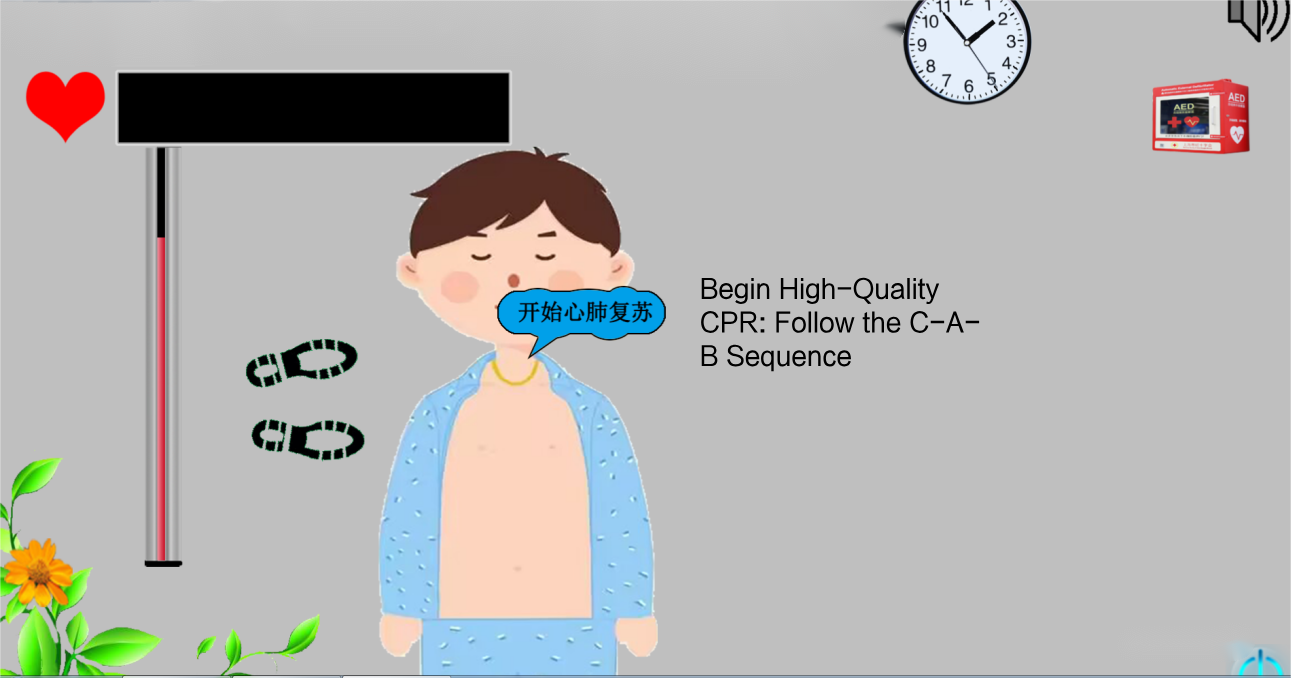
**


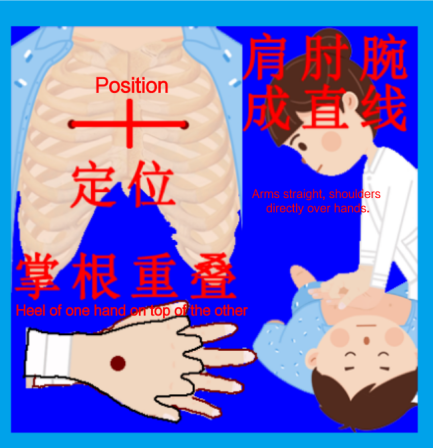

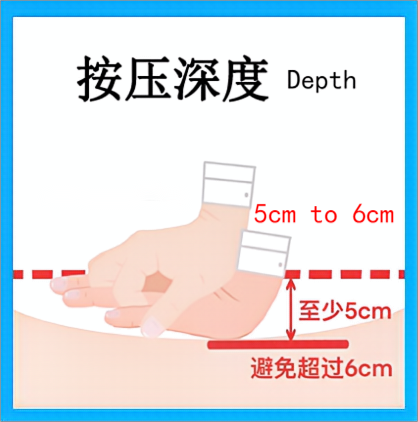

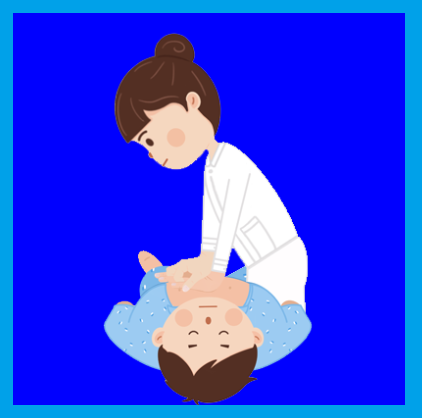

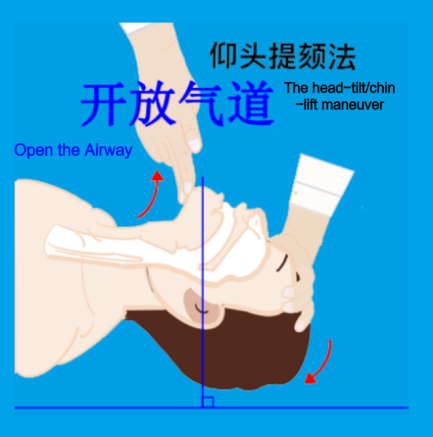

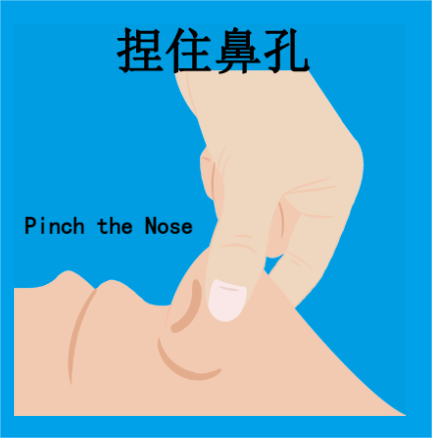

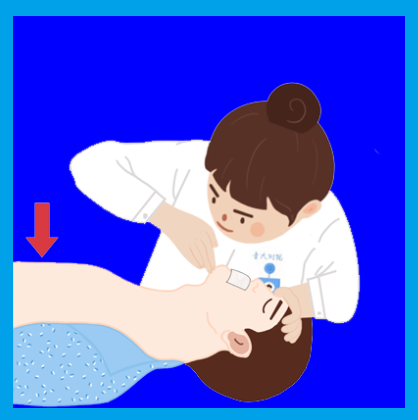


**Early Defibrillation with AED**

**
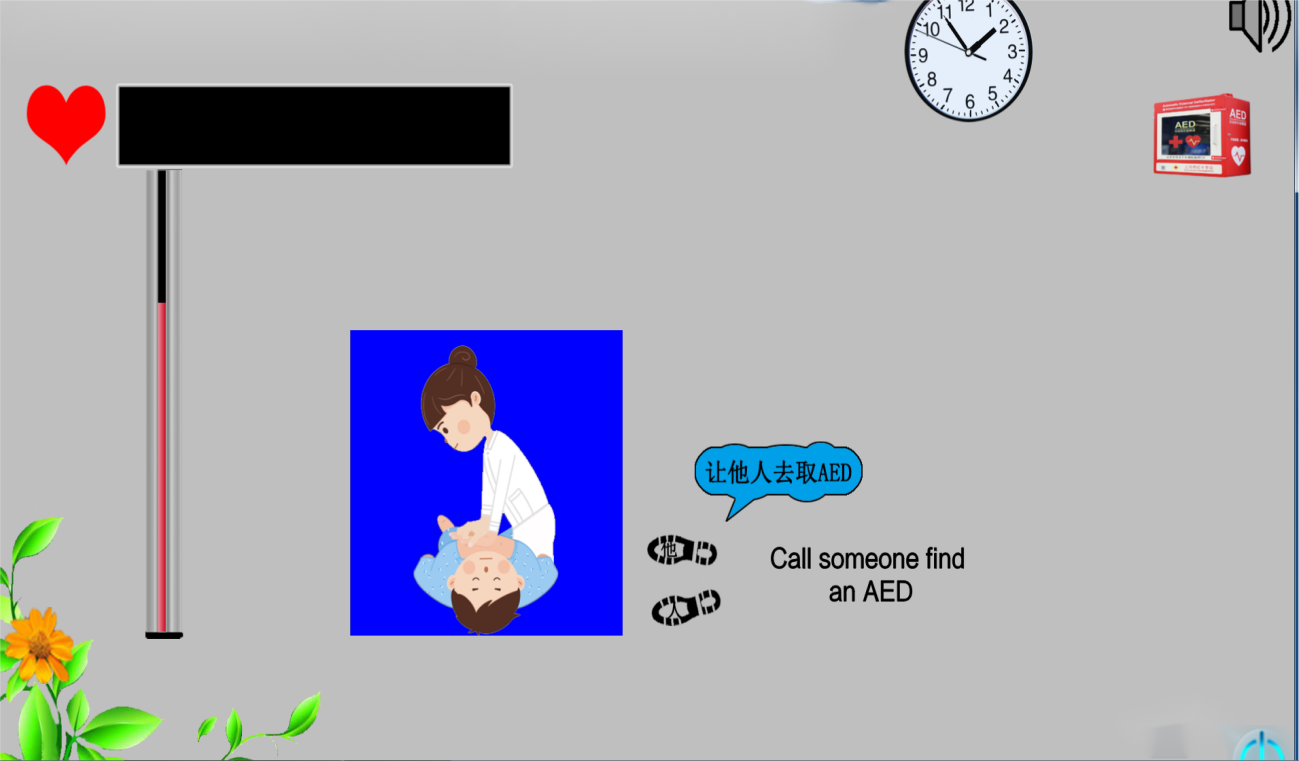
**

**
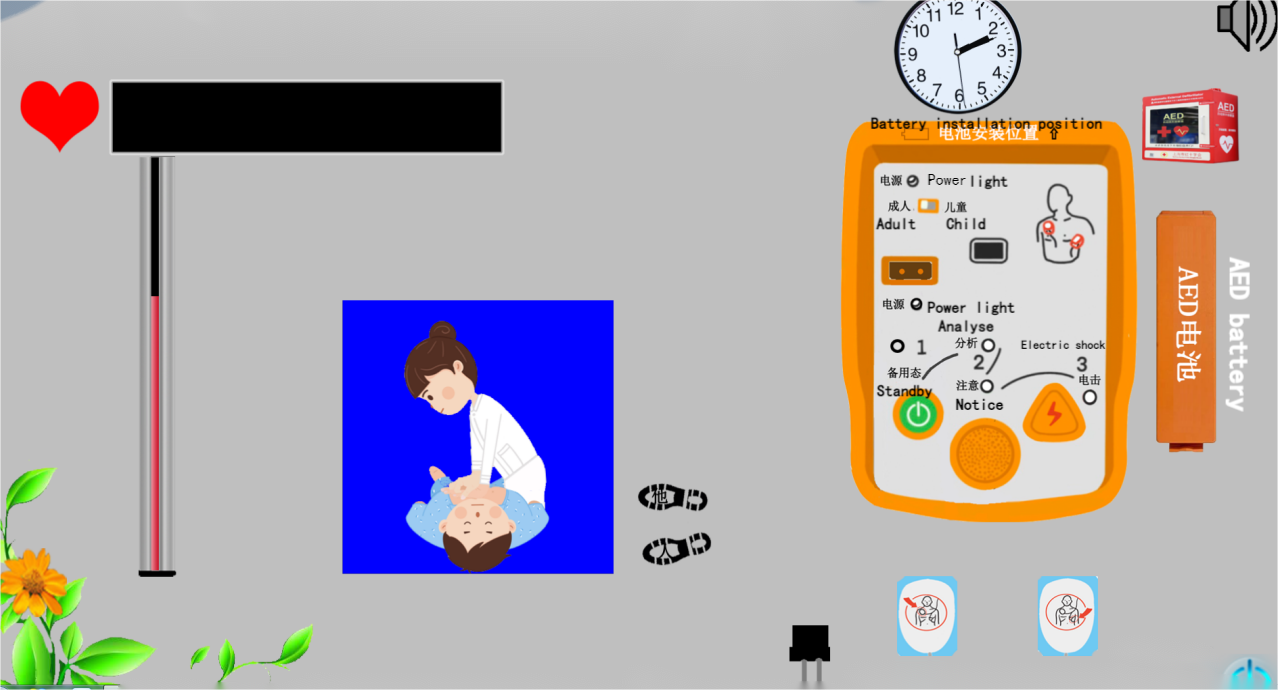
**

**
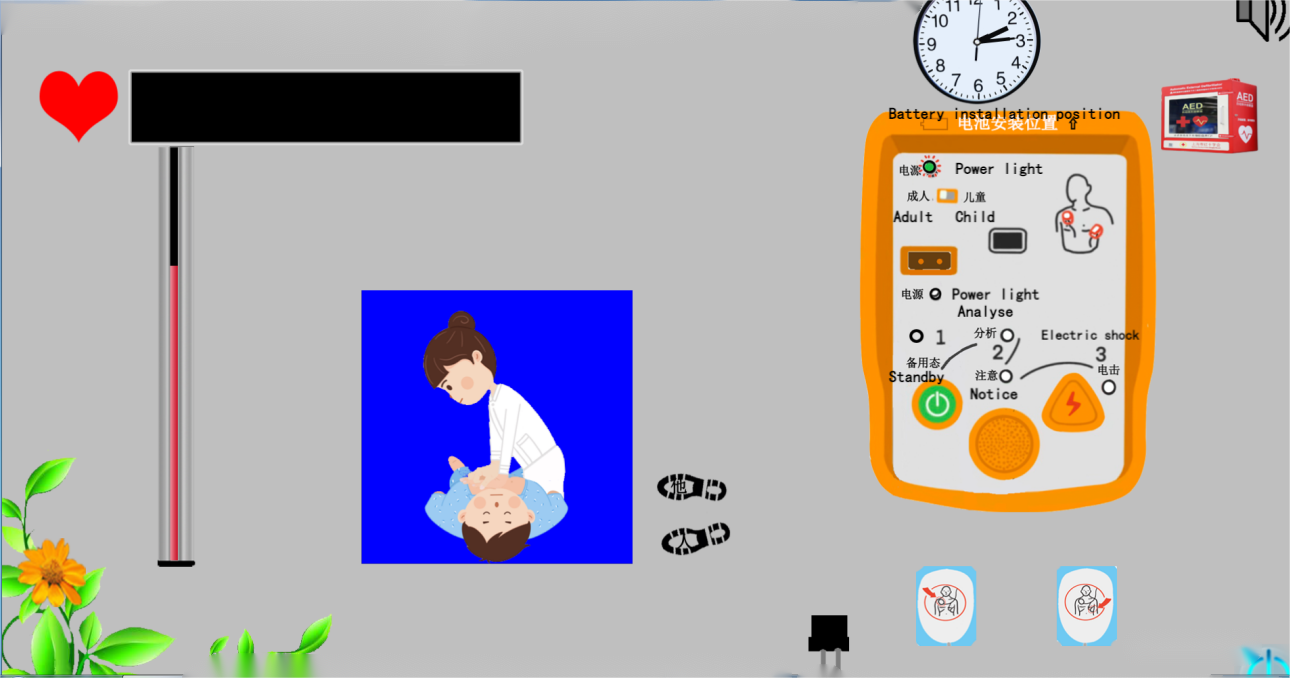
**

**
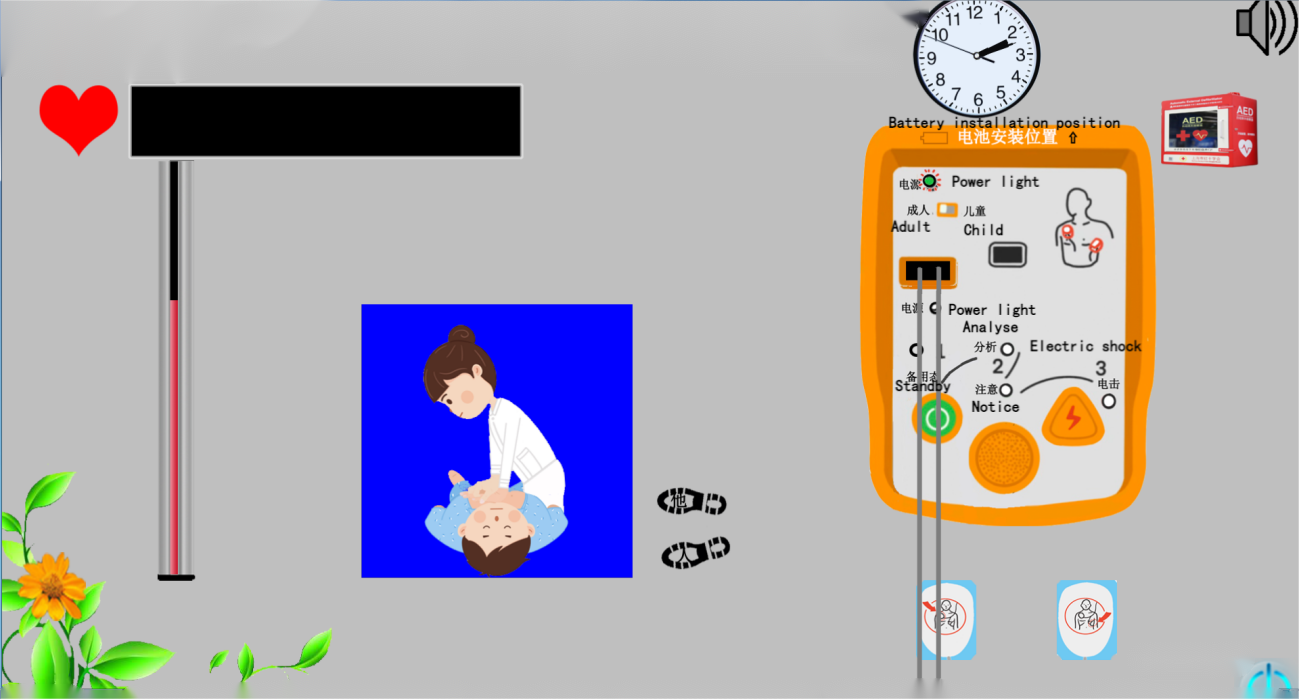
**

**
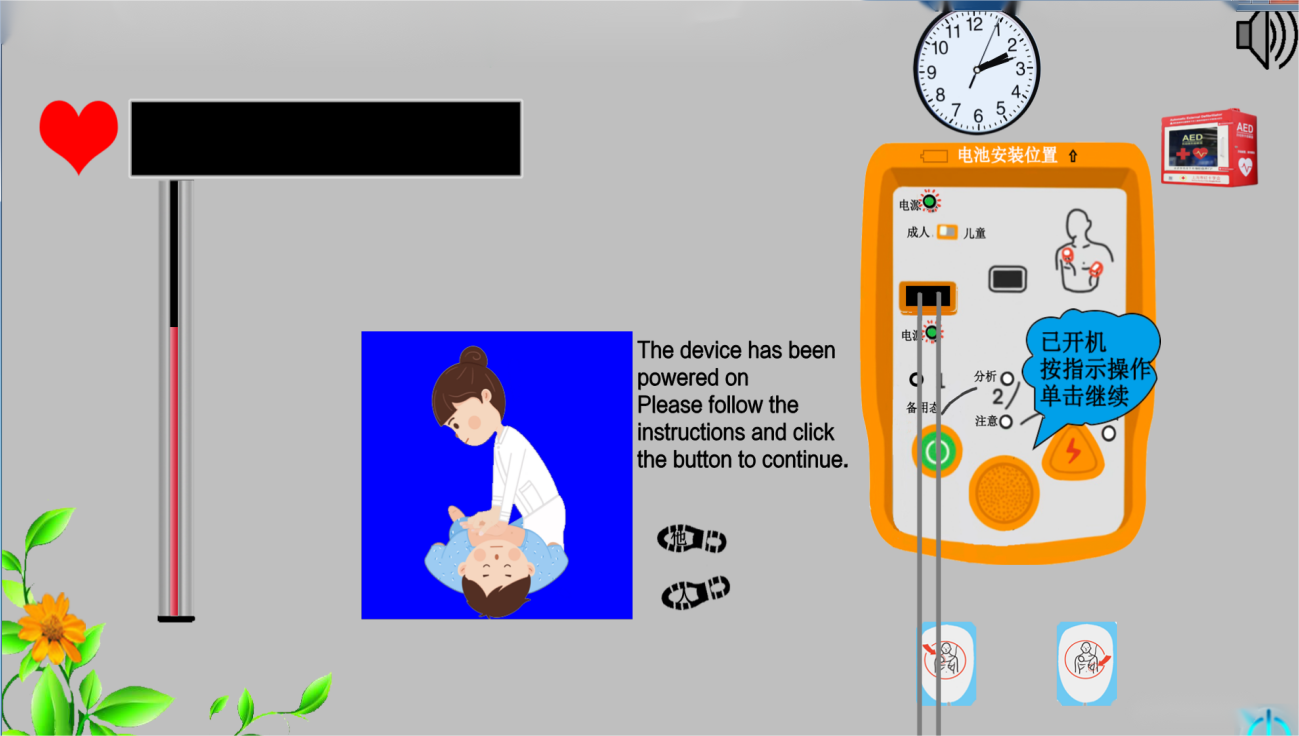
**

**
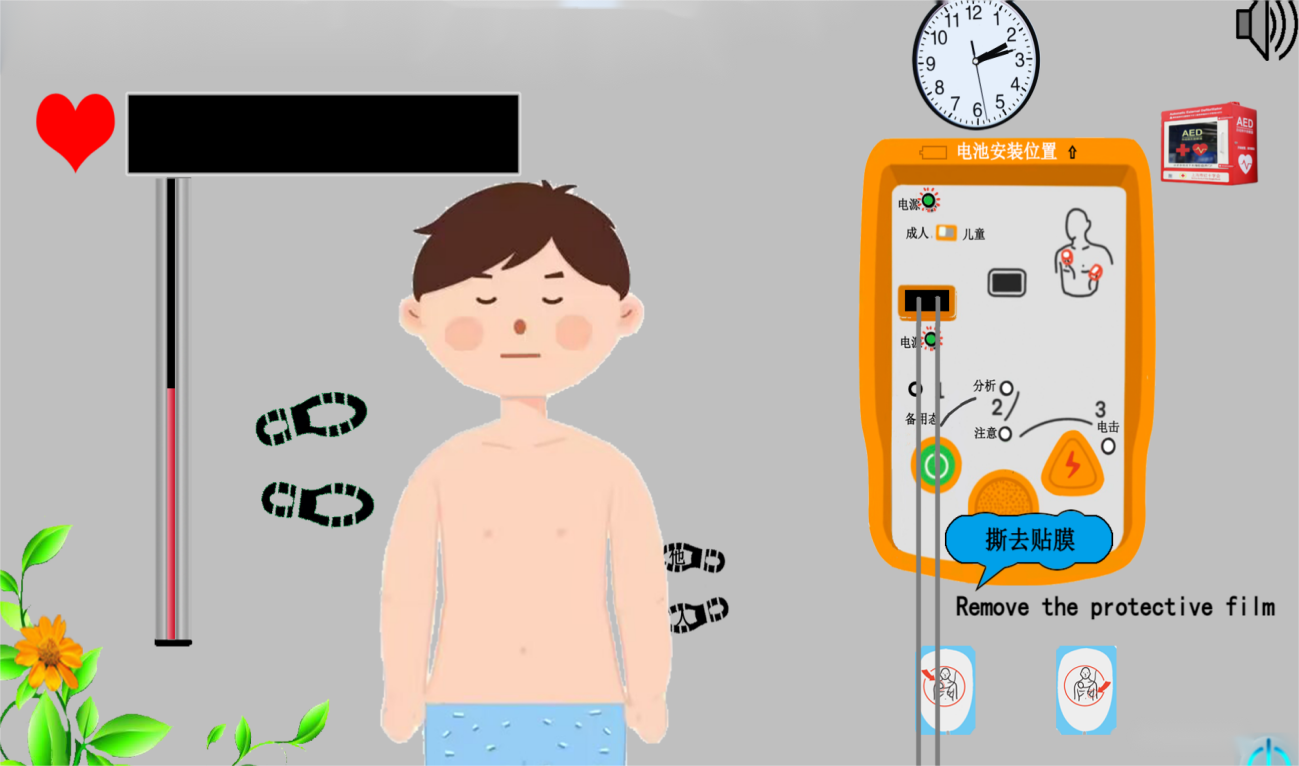
**

**
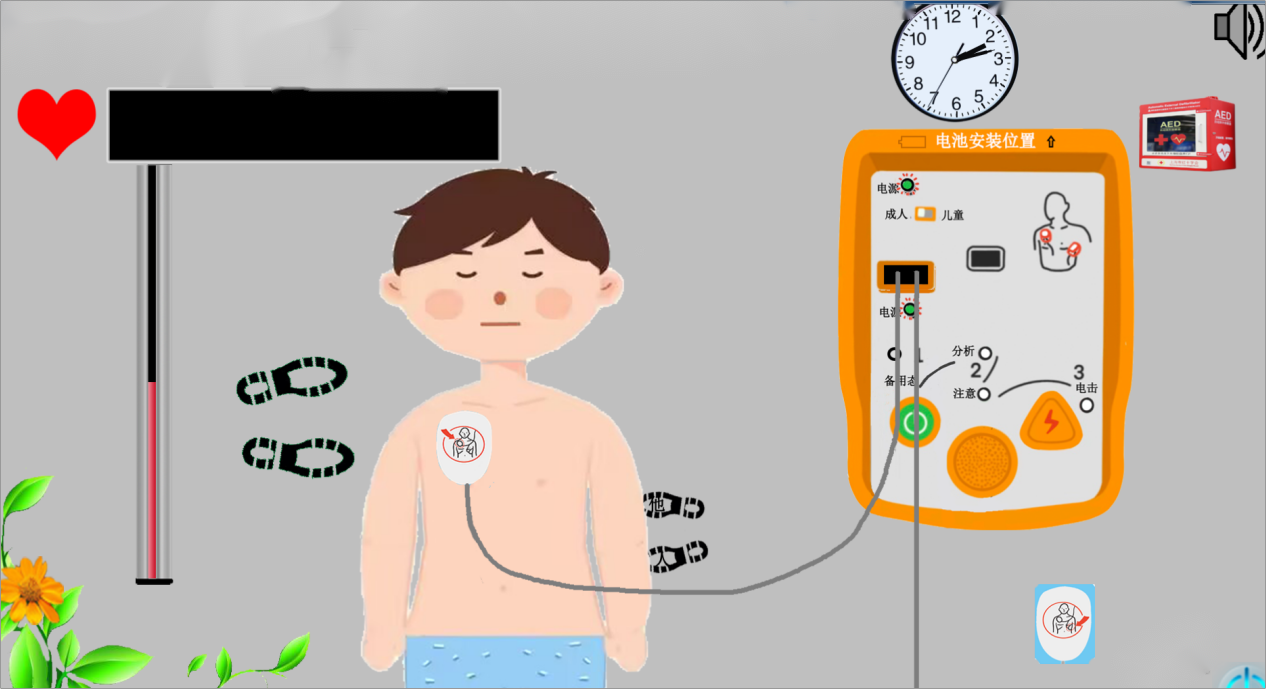
**

**
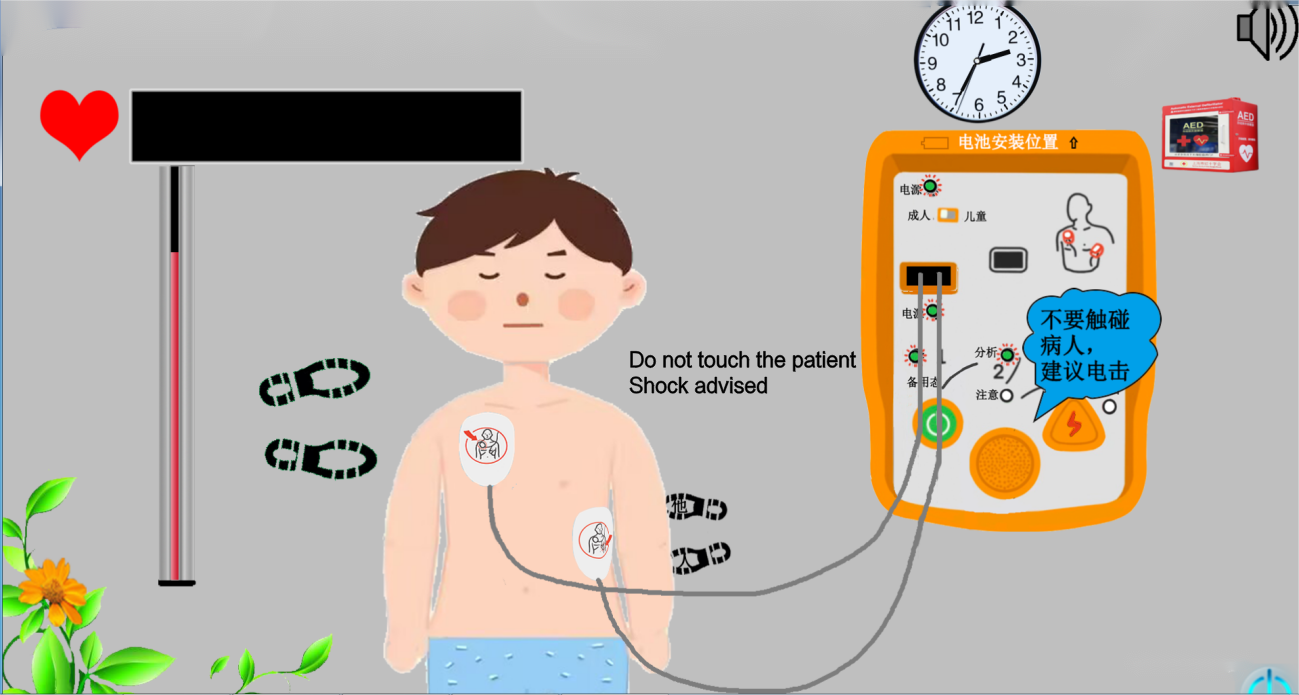
**

**
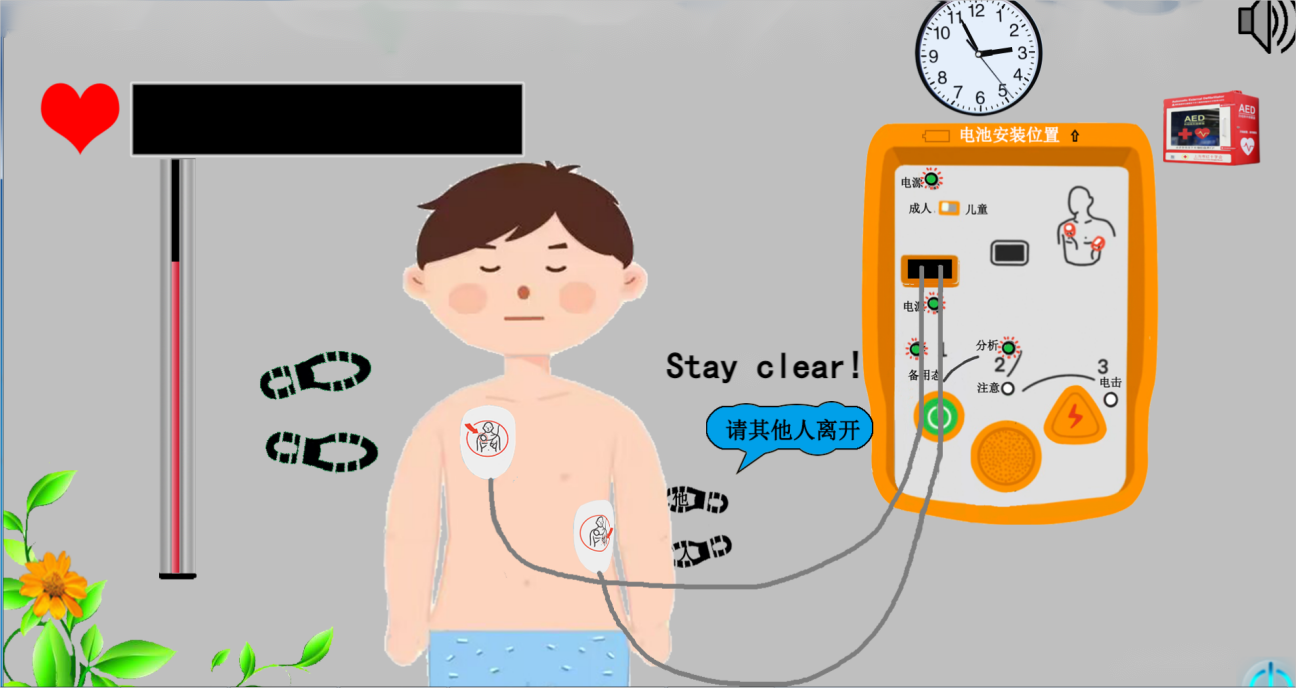
**

**
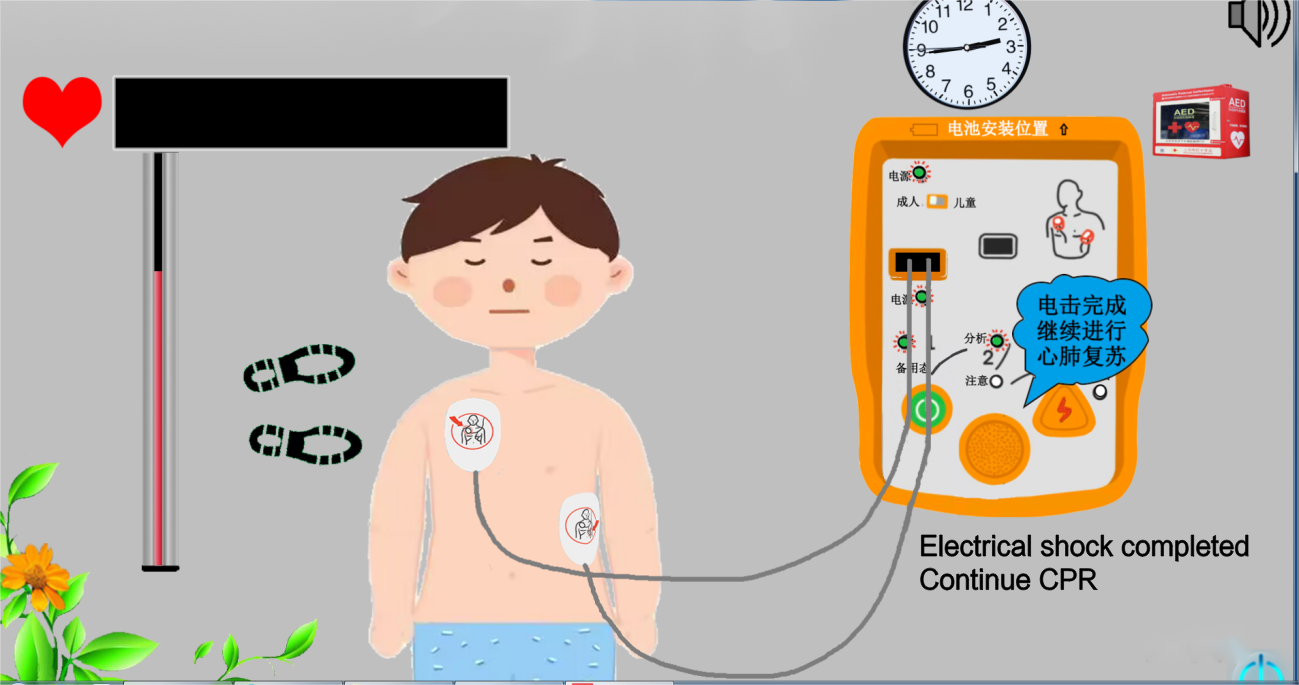
**

- **Practice Results Screenshots**


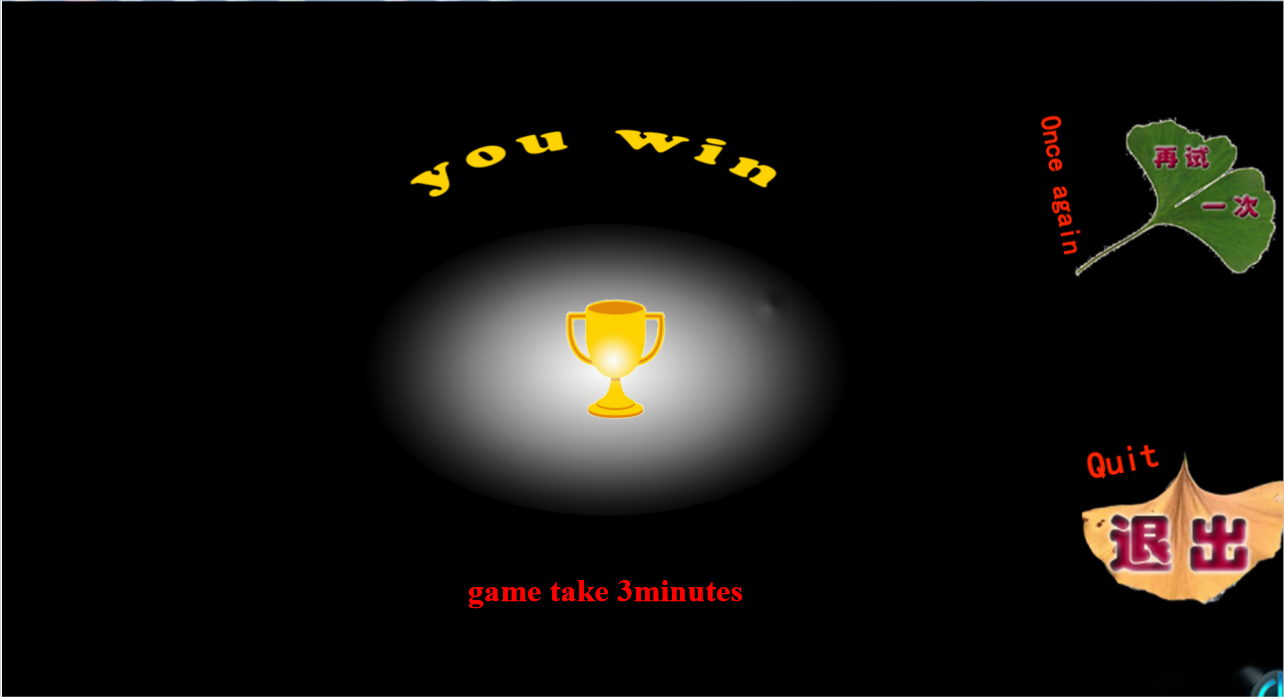


**
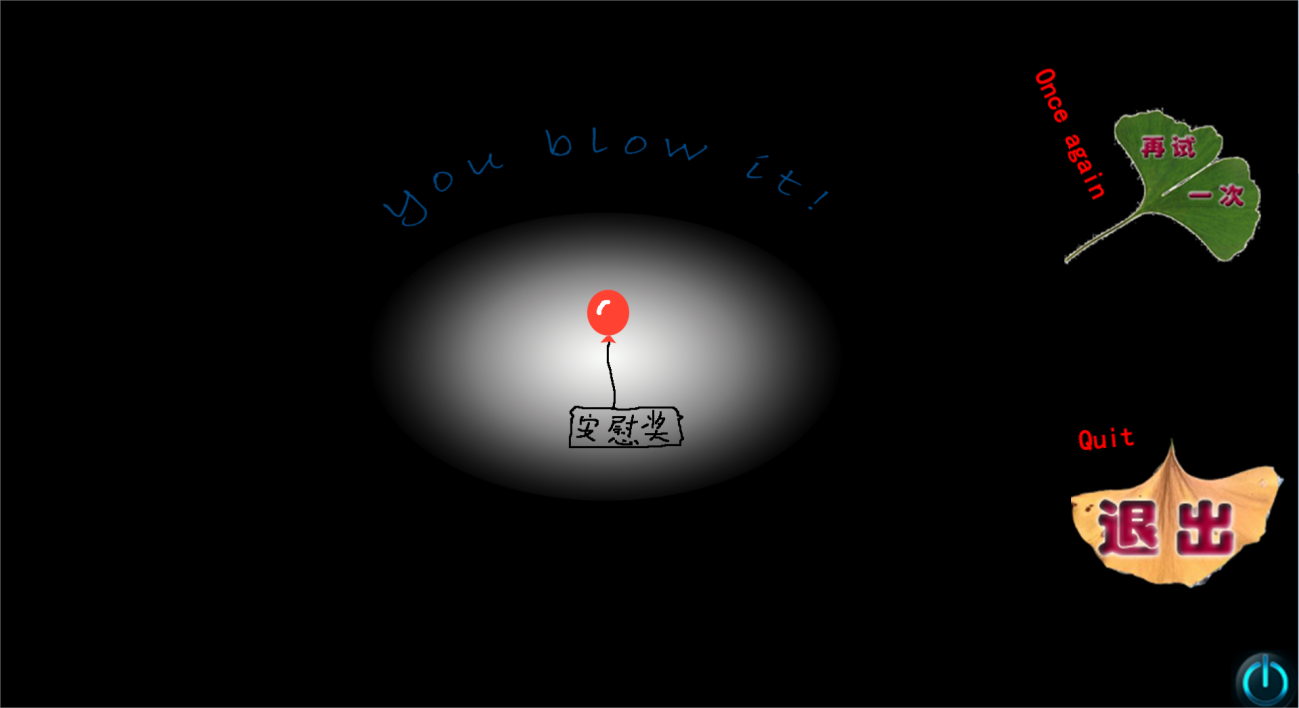
**
